# Supplementary material for: Motility and phototaxis of Gonium, the simplest differentiated colonial alga
Source: Phys Rev E. Author manuscript; Available in PMC 2024 Jun 11. (PMC7616084; doi:10.1103/PhysRevE.101.022416)
Supplement: Appendix [file EMS196593-supplement-Appendix.pdf]

APPENDIX A: MATERIALS AND METHODS

Data underlying the figures in this paper are available in the Supplemental Material [29].

1. Culture conditions

Wild-type *Gonium pectorale* colonies (strain CCAC 3275 B from the Cologne Biocenter) were grown in standard *Volvox* medium in an incubator (Binder) at 24° under 3800 lux illumination in a 14:10h light-dark cycle.

2. Observation of swimming

Chambers were prepared using two glass slides, sealed with Frame-Seal Incubation Chambers (SLF0201, 9 × 9 mm, BIO-RAD Laboratories). They were subsequently mounted on a Nikon TE2000-U inverted microscope for observation with a 4× or 10× Nikon Plan Fluor objective. Nonphototactic red illumination of the samples was achieved with a long-pass filter (620 nm) in the light path. Observations were recorded with a high-speed video camera (Phantom V311; see also below) at 30 fps. A pair of blue LEDs (M470L2, Thorlabs), connected to LED drivers (LEDD1B, Thorlabs), were placed on either sides of the chamber, and separately controlled, as described in Fig. 2(a). To assure maximum photoresponse, the wavelength of 470 nm was chosen to be close to the absorption maximum rhodopsin [41], the protein in the eyespot responsible for light detection [14].

3. Micropipette experiments

We used borosilicate glass pipettes (Sutter Instruments, outer diameter 1.0 mm, inner diameter 0.75 mm), a pipette puller (P-97, Sutter Instruments) and a multifunction microforge controller (DMF1000, World Precision Instruments) to produce micropipettes of inner diameter ~20 μm, with polished edges.

Chambers were fabricated by gluing short spacers to glass slides with UV setting glue (NOA61), with UV exposure for 1 min (ELC-500 lamp, Electro-Lit Corporation), leaving apertures at 90° for the micropipette and the optical fiber to enter the chamber, as sketched in Fig. 2(b). Colonies were caught on the micropipette, through which fluid was gently aspirated via a 10-ml syringe (BD Luer-Lok 305959). The optical fiber (FT400EMT, Thorlabs) was connected to a 470-nm LED (M470F3, Thorlabs) and driver (DC2200, Thorlabs), enabling intensity and timing control. We took care that the fiber end enters the liquid in the chamber to avoid losses of light by reflection at the air-water interface and that it is aligned with the tip of the micropipette. Synchronization of the light source to the camera was made via a NI-DAQ (BNC-2110, National Instruments). Light intensities were measured with a lux meter (Lutron LX-101). We verified proportionality

between the intensity (in lux) from of the optical fiber and the current (mA) provided by the driver, to extrapolate light intensities below 1 lux, where the meter saturates.

PIV experiments are conducted by adding nonfluorescent beads (Polybead Polystyrene Cat. 07310, Polysciences), of diameter 1 μm, to the *Gonium* suspension. Image acquisition was performed on a similar microscope with a 20× Plan Fluor (Nikon) or a 63× water immersion Plan Apochromat objective (Zeiss) with a 45-60 Nikon adapter, connected to the same video-camera recording at 200 fps. Image analysis was performed via the Matlab tool PIVlab, with a window size adapted to the chosen microscope objective to ensure the presence of at least three to five particles.

APPENDIX B: INFERRING HELICAL MOTION FROM PLANAR PROJECTIONS

We define three velocities for a helical trajectory: the "mean velocity" $v_m = \lambda/T$, where T is the time to cover one wavelength λ, the true (3D) instantaneous velocity v_{3D}, and the apparent (2D) instantaneous velocity $v_{2D} = v$. From microscopy, we access v_m and v, from which we deduce v_{3D}. To relate these velocities, we rewrite the helical trajectory in parametric form:

$$(x(z), y(z)) = A(\cos kz, \sin kz), \tag{B1}$$

where $k = 2\pi/\lambda$. The instantaneous velocity is $v_{3D} = l/T$, where $l = \int_0^\lambda ds$ is the curved length covered by *Gonium*. In 3D, we have $ds = dz\sqrt{1 + x_z^2 + y_z^2}$, yielding

$$\begin{aligned} v_{3D} &= \frac{1}{T}\int_0^\lambda \sqrt{1 + (kA)^2} dz \\ &= \frac{\lambda}{T}\sqrt{1 + \tan^2\chi} = \frac{v_m}{\cos\chi}. \end{aligned} \tag{B2}$$

If we assume that the 2D plane of view is (x, z), then for the apparent (2D) instantaneous velocity v we now have $ds = dz\sqrt{1 + x_z^2}$, so

$$v_{2D} = \frac{1}{T}\int_0^\lambda \sqrt{1 + \tan^2\chi \sin^2(kz)} dz, \tag{B3}$$

which is an elliptic integral of the second kind of parameter $\tan^2\chi$. For small χ, a Taylor expansion gives $v_m/v_{3D} \simeq 1 - \chi^2/2$ and $v_m/v_{2D} \simeq 1 - \chi^2/4$. For the typical values found for *Gonium*, $\chi \approx 30^\circ$, we deduce that $v_{2D}/v_{3D} \simeq 0.93$ and therefore conclude that approximating the instantaneous velocity by its 2D projection is reasonable.

APPENDIX C: HELICAL TRAJECTORIES

We relate here the pitch angle χ of the helical trajectories to the uneven distribution of flagellar forces. The geometry is shown in Fig. 1(d), with $\hat{\mathbf{e}}_3$ the symmetry axis of the *Gonium* body. In this geometry, the rotation vector $\mathbf{\Omega}$ is along the mean swimming direction $\hat{\mathbf{e}}_z$. The pitch angle χ is the angle between the instantaneous velocity $\mathbf{U}$ and $\hat{\mathbf{e}}_z$. The symmetry axis of the *Gonium* body describes a cone around z of constant apex angle ζ, with $\zeta < \chi$ because of the anisotropic resistance matrices. The Euler angles for this geometry are $\theta = \zeta$, $\dot{\phi} = \omega_3/\cos\zeta$, and $\dot{\psi} = 0$. The frictional torque Eq. (1b) is thus

$$\mathbf{L}_v = -\eta R^3 \begin{bmatrix} l_1\dot{\phi}\sin\zeta\sin\psi \\ l_1\dot{\phi}\sin\zeta\cos\psi \\ l_3(\dot{\psi} + \dot{\phi}\cos\zeta) \end{bmatrix}. \tag{C1}$$

The simplest defect compatible with such a helical trajectory is a modulation of the axial force developed by the peripheral flagella in the form [cf. Eq. (5)]

$$\mathbf{f}_p(\alpha) = \mathbf{f}_p^{(0)}(1 + \xi\cos\alpha), \tag{C2}$$

with ξ an "imbalance" parameter. We assume here that β does not depend on α, so that the defect affects the peripheral and axial components $f_{p\parallel}$ and $f_{p\perp}$ in the same way. From this force distribution, the total force and torque (3) are

$$\mathbf{F} = \left[F_c + 2\pi f_{p\parallel}^{(0)}\right]\hat{\mathbf{e}}_3 + \pi\xi f_{p\perp}^{(0)}\hat{\mathbf{e}}_2, \tag{C3}$$

$$\mathbf{L} = 2\pi R f_{p\perp}^{(0)}\hat{\mathbf{e}}_3 - \pi\xi R f_{p\parallel}^{(0)}\hat{\mathbf{e}}_2. \tag{C4}$$

Since the force and the torque now have components along $\hat{\mathbf{e}}_2$, the velocity and angular velocity do as well. The phase choice in Eq. (5) assigns maximum flagella force along $\hat{\mathbf{e}}_1$, and implies $L_1 = 0$, so $\psi = 0$ in Eq. (C1).

To compute the angle ζ, we first solve for the torque-angular velocity relation along $\hat{\mathbf{e}}_2$ and $\hat{\mathbf{e}}_3$,

$$-\eta R^3 l_1\dot{\phi}\sin\zeta = -\pi\xi R f_{p\parallel}^{(0)}, \tag{C5}$$

$$-\eta R^3 l_3\dot{\phi}\cos\zeta = 2\pi R f_{p\perp}^{(0)}. \tag{C6}$$

Solving for ζ yields

$$\tan\zeta = \xi\frac{l_3}{2l_1}\tan\beta \tag{C7}$$

with $\tan\beta = -f_{p\parallel}^{(0)}/f_{p\perp}^{(0)}$. A symmetric *Gonium* ($\xi = 0$) naturally has $\zeta = 0$.

The helix pitch angle χ, the angle between the instantaneous velocity $\mathbf{U}$ and the mean swimming direction $\hat{\mathbf{e}}_z$, is found by solving the velocity-force relation along $\hat{\mathbf{e}}_2$ and $\hat{\mathbf{e}}_3$,

$$\eta R k_1 U_2 = \pi\xi f_{p\perp}^{(0)}, \tag{C8}$$

$$\eta R k_3 U_3 = F_c + 2\pi f_{p\parallel}^{(0)}. \tag{C9}$$

The angle ζ' between $\hat{\mathbf{e}}_3$ and $\mathbf{U}$ is

$$\tan\zeta' = \frac{U_2}{U_3} = \xi\frac{k_3}{2k_1}\frac{1}{\tan\beta}\frac{1}{1 + F_c/\left(2\pi f_{p\parallel}^{(0)}\right)}. \tag{C10}$$

We note that the vectors $\mathbf{\Omega}$ ($\parallel \hat{\mathbf{e}}_z$), $\hat{\mathbf{e}}_3$, and $\mathbf{U}$ are in the same plane ($\hat{\mathbf{e}}_2, \hat{\mathbf{e}}_3$). The total angle χ between $\hat{\mathbf{e}}_z$ and $\mathbf{U}$ is therefore simply $\chi = \zeta + \zeta'$. For small ξ, we have $\tan\chi \simeq \tan\zeta + \tan\zeta'$, yielding Eq. (6),

$$\tan\chi \simeq \frac{\xi}{2}\left\{\frac{l_3}{l_1}\tan\beta + \frac{k_3}{k_1}\frac{1}{\tan\beta}\frac{1}{1 + F_c/\left[2\pi f_{p\parallel}^{(0)}\right]}\right\}. \tag{C11}$$

We can rewrite this relation using the discret point-force model. Assuming that all the individual flagellar forces F_i are identical, we have $F_c = 8F_i$ and $2\pi f_{p\parallel}^{(0)} = 24F_i\sin\beta$,

yielding

$$\tan\chi \simeq \frac{\xi}{2}\left[\frac{l_3}{l_1}\tan\beta + \frac{k_3}{k_1}\frac{1}{\tan\beta + 1/(3\cos\beta)}\right]. \quad \text{(C12)}$$

The helix parameters A and λ are derived by expressing the velocity in the laboratory frame,

$$U_x(t) = -(U_2\cos\zeta - U_3\sin\zeta)\sin(\dot\phi t), \quad \text{(C13a)}$$

$$U_y(t) = (U_2\cos\zeta - U_3\sin\zeta)\cos(\dot\phi t), \quad \text{(C13b)}$$

$$U_z = U_2\sin\zeta + U_3\cos\zeta. \quad \text{(C13c)}$$

with $\dot\phi = \omega_3/\cos\zeta$. Here U_z corresponds to v_m, the mean velocity along z. Integrating these equations in time yields the helix amplitude and wavelength,

$$A = \frac{U_3\sin\zeta - U_2\cos\zeta}{\dot\phi}, \quad \text{(C14a)}$$

$$\lambda = 2\pi\frac{U_2\sin\zeta + U_3\cos\zeta}{\dot\phi}. \quad \text{(C14b)}$$

Using $\tan\chi = 2\pi A/\lambda$, we obtain

$$\tan\chi = \frac{U_3\tan\zeta - U_2}{U_3 + U_2\tan\zeta}, \quad \text{(C15)}$$

which can be shown to be equivalent to Eq. (C12) to first order in ξ.

APPENDIX D: NUMERICAL METHODS

Here we give details of the numerical techniques and show characteristic values extracted from the computed wavy trajectories. We use the geometry shown in Fig. 4(b) and described in the text. Typical Reynolds numbers for swimming *Gonium* are $\sim 10^{-3}$ in the Stokes regime. The velocity of the surrounding fluid at a position $\mathbf{x}$ can therefore be expressed as boundary integrals,

$$\mathbf{u}(\mathbf{x}) = -\frac{1}{8\pi\mu}\int_{\substack{\text{cell}\\\text{body}}}\mathbf{J}(\mathbf{x},\mathbf{x}')\cdot\mathbf{q}(\mathbf{x}')\,dA(\mathbf{x}') - \frac{1}{8\pi\mu}\sum_{j=1}^{32}\int_{\text{flagella}}[\mathbf{J}(\mathbf{x},\mathbf{x}') + \mathbf{W}(\mathbf{x},\mathbf{x}')]\cdot\mathbf{f}(\mathbf{x}')\,dl_j(\mathbf{x}') - \frac{1}{8\pi\mu}\sum_{j=1}^{32}\mathbf{J}(\mathbf{x},\mathbf{x}')\cdot\mathbf{F}_j(\mathbf{x}'). \quad \text{(D1)}$$

Here $\mathbf{J}$ is the Stokeslet kernel, $\mathbf{W}$ the kernel for slender body theory, $\mathbf{q}$ the traction force on the cell body, $\mathbf{f}$ the traction force on flagella, and $\mathbf{F}$ the point force generated by the flagella. The first term of the right-hand side of Eq. (D1) represents the drag of the cell body, which is solved by a boundary element method with a mesh of 320 triangles. The second term accounts for the drag of the 32 flagella, which is solved by slender body theory with 10 slender elements per flagellum. The third term represents thrust and spin forces generated by flagella. A no-slip velocity boundary condition is applied on the cell body and along the flagella; force- and torque-free conditions are applied to the whole body. Detailed functional forms and the velocity along the flagella can be found in Itoh *et al.* [35]; here we solve Eq. (D1) in a similar manner.

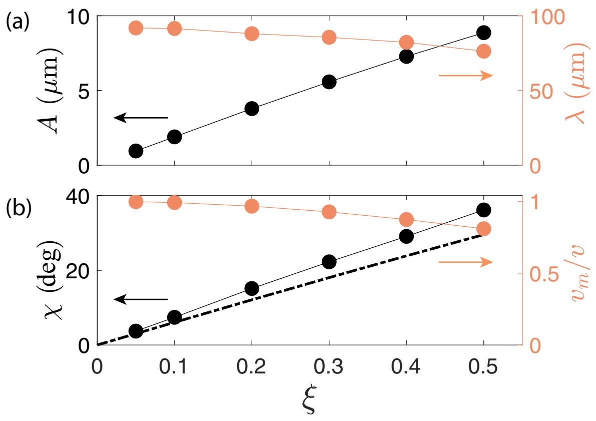

FIG. 13. Properties of wavy helical trajectories. (a) Amplitude A of the oscillations and wavelength λ of the helix as a function of the defect parameter ξ. (b) Pitch angle χ and swimming efficiency v_m/v as a function of the imbalance parameter ξ. The dashed line shows the theoretical prediction for χ as a function of ξ, obtained from Eq. (6).

The amplitude A of the oscillations of the computed wavy trajectories, using the force variation around *Gonium* given by Eq. (5), linearly increases with the strength ξ of the imbalance (Fig. 13), while the wavelength λ is hardly impacted. The deduced pitch angle χ therefore also linearly increases with ξ, in good agreement with Eq. (6), plotted by the dashed line. This enables us to deduce the experimental imbalance amplitude, as $\chi \simeq 30^\circ$ corresponds to $\xi \simeq 0.4$. Finally, the decrease in swimming efficiency v_m/v follows the geometrical prediction $v_m/v = \cos\chi$, as plotted in Fig. 3(c).

APPENDIX E: MAXIMUM OF THE ADAPTIVE RESPONSE

Defining $x = t/\tau_r$ in the step response (9) for $t > 0$ we have

$$p_{\text{step}}(x) = \frac{\mu s_0}{1-\rho}(e^{-\rho x} - e^{-x}), \quad \text{(E1)}$$

where again $\rho = \tau_r/\tau_a$. The maximum response obtained by setting $dp_{\text{step}}/dx = 0$ occurs at $x^* = -(1-\rho)^{-1}\ln\rho$, with the magnitude $p^*_{\text{step}} = p_{\text{step}}(x^*)$ given by the amusing function

$$p^*_{\text{step}} = \rho^{\rho/(1-\rho)}. \quad \text{(E2)}$$

As shown in Fig. 14, this has a maximum at $\rho = 0$, confirming the statement in Sec. III C that the maximum amplitude response is found in the limit $\tau_r/\tau_a \to 0$.

APPENDIX F: PHOTOTACTIC GAIN FUNCTION

We estimate here the photoresponse p_α at an arbitrary angle α around *Gonium*. This allows computation of the phototactic torque (3) from the flagella force (7). The balance with the viscous torque eventually leads to the phototactic reorientation trajectory. This implies the definition of a gain function $\mathcal{G}$. From (10), the response to light stimulation $s(t)$ is

$$p(t) = \frac{\mu}{1-\rho}\int_{-\infty}^{t} s(t')\left[\frac{e^{-(t-t')/\tau_r}}{\tau_r} - \frac{e^{-(t-t')/\tau_a}}{\tau_a}\right]dt'. \quad \text{(F1)}$$

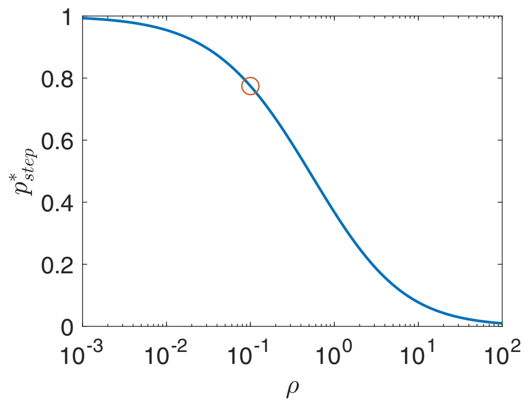

FIG. 14. Maximum amplitude (E2) of the adaptive phototactic response to a step-up in light intensity, as a function of the ratio $\rho = \tau_r/\tau_a$. Red circle indicates experimental average.

In this model, $p(t)$ has a slight negative overshoot when $s(t)$ decreases. However, for $\tau_r \ll \tau_a$, one has $p(t) > 0$ for almost all time, and the integration of p among the flagella on the illuminated side remains positive.

To evaluate the integral, we use the following geometry. We consider that only flagella on the illuminated side of the *Gonium*, defined by $\alpha = -\pi/2 \cdots \pi/2$, contribute to the reorientation torque. A flagellum at angle α has traveled from $-\pi/2$ to α at constant spin ω_3. After its transit to the dark side, each flagellum looses the memory of its previous transit on the illuminated side, so that $p = 0$ when it reaches $\alpha = -\pi/2$. With $s(\alpha) = s_0 \cos\phi \cos\alpha$ and using $\alpha = \omega_3 t$, Eq. (F1) becomes

$$p_\alpha = \frac{\mu s_0 \cos\phi}{1-\rho}[I(\omega_3\tau_r, \alpha) - I(\omega_3\tau_a, \alpha)], \quad \text{(F2)}$$

with

$$I(A, \alpha) = \frac{1}{A}\int_{-\pi/2}^{\alpha} \cos\alpha' \, e^{-(\alpha-\alpha')/A} \, d\alpha' \quad \text{(F3)}$$

$$= \frac{1}{A^2+1}[(A\sin\alpha + \cos\alpha) + Ae^{-(\alpha+\pi/2)/A}]. \quad \text{(F4)}$$

We finally compute the torque (3) from the flagella force (7). Because of the delay induced by the adaptive response, the torque is no longer along $\hat{\mathbf{e}}_z$, implying a change in θ. Since we assumed $\theta = \pi/2$ earlier, we consistently neglect this effect and consider only the z component of the torque,

$$L_z = Rf_{p\parallel}^{(0)} \int_{-\pi/2}^{\pi/2} p_\alpha \cos\alpha \, d\alpha. \quad \text{(F5)}$$

Inserting (F2), we rewrite this integral as

$$L_z = Rf_{p\parallel}^{(0)} \frac{\mu s_0 \cos\phi}{1-\rho}[J(\omega_3\tau_r) - J(\omega_3\tau_a)], \quad \text{(F6)}$$

where

$$J(A) = \int_{-\pi/2}^{\pi/2} I(A, \alpha)\cos\alpha \, d\alpha$$

$$= \frac{1}{A^2+1}\left[\frac{\pi}{2} + \frac{A^3}{A^2+1}(1+e^{-\pi/A})\right]. \quad \text{(F7)}$$

We have $J(0) = \pi/2$ and $J(A) \simeq 2/A$ for $A \gg 1$. Balancing this torque with the z component of the friction torque $L_{vz} = -\eta R^3 l_1 \dot{\phi}$ yields a differential equation in the form (14), from which we identify the gain function

$$\mathcal{G}(\omega_3\tau_r, \omega_3\tau_a) = \frac{2}{\pi}\frac{[J(\omega_3\tau_r) - J(\omega_3\tau_a)]}{1-\rho}. \quad \text{(F8)}$$
